# d-Amino acids differentially trigger an inflammatory environment in vitro

**DOI:** 10.1007/s00726-023-03360-8

**Published:** 2024-02-03

**Authors:** Siew Hwei Yap, Cheng Siang Lee, Nur Diyana Zulkifli, Darshinie Suresh, Kenji Hamase, Kumitaa Theva Das, Reena Rajasuriar, Kok Hoong Leong

**Affiliations:** 1 Centre of Excellence for Research in AIDS (CERiA), Universiti Malaya, Kuala Lumpur, Malaysia; 2 Department of Medicine, Faculty of Medicine, Universiti Malaya, Kuala Lumpur, Malaysia; 3 Department of Biomedical Sciences, Advanced Medical and Dental Institute, Universiti Sains Malaysia, Kepala Batas, Malaysia; 4 Department of Biological Sciences and Biotechnology, Faculty of Science and Technology, Universiti Kebangsaan Malaysia, Bangi, Malaysia; 5 Graduate School of Pharmaceutical Sciences, Kyushu University, Fukuoka, Japan; 6 Peter Doherty Institute for Infection and Immunity, Melbourne University, Melbourne, VIC Australia; 7 Department of Pharmaceutical Chemistry, Faculty of Pharmacy, Universiti Malaya, Kuala Lumpur, Malaysia

**Keywords:** d-Amino acid oxidase, d-Serine, d-Alanine, Inflammation, TNF-α

## Abstract

Studies in vivo have demonstrated that the accumulation of d-amino acids (d-AAs) is associated with age-related diseases and increased immune activation. However, the underlying mechanism(s) of these observations are not well defined. The metabolism of d-AAs by d-amino oxidase (DAO) produces hydrogen peroxide (H_2_O_2_), a reactive oxygen species involved in several physiological processes including immune response, cell differentiation, and proliferation. Excessive levels of H_2_O_2_ contribute to oxidative stress and eventual cell death, a characteristic of age-related pathology. Here, we explored the molecular mechanisms of d-serine (d-Ser) and d-alanine (d-Ala) in human liver cancer cells, HepG2, with a focus on the production of H_2_O_2_ the downstream secretion of pro-inflammatory cytokine and chemokine, and subsequent cell death. In HepG2 cells, we demonstrated that d-Ser decreased H_2_O_2_ production and induced concentration-dependent depolarization of mitochondrial membrane potential (MMP). This was associated with the upregulation of activated NF-кB, pro-inflammatory cytokine, TNF-α, and chemokine, IL-8 secretion, and subsequent apoptosis. Conversely, d-Ala-treated cells induced H_2_O_2_ production, and were also accompanied by the upregulation of activated NF-кB, TNF-α, and IL-8, but did not cause significant apoptosis. The present study confirms the role of both d-Ser and d-Ala in inducing inflammatory responses, but each via unique activation pathways. This response was associated with apoptotic cell death only with d-Ser. Further research is required to gain a better understanding of the mechanisms underlying d-AA-induced inflammation and its downstream consequences, especially in the context of aging given the wide detection of these entities in systemic circulation.

**Supplementary Information:**

The online version contains supplementary material available at 10.1007/s00726-023-03360-8.

## Introduction

Amino acids exist naturally in l- and d-forms, as they have a chiral center, α-carbon. L-amino acids have long been known for ribosome-based protein synthesis in all mammals. However, it was only a decade ago that d-amino acids (d-AAs) were discovered to be present in mammals, owing to the advancement in the detection methods for chiral amino acids. Since then, d-AAs have been implicated in various biological processes, where each entity appears to play a different and specialized role. For instance, D-aspartate is a major regulator of adult neurogenesis, and d-serine (d-Ser) acts as a co-agonist of the *N*-methyl d-aspartate-type (NMDA) glutamate receptors in the brain, which are involved in mammalian learning, memory, and behaviour (Errico et al. 2008; Mothet et al. 2000). Additionally, d-AAs have been found to accumulate in tissues and organs in age-related diseases, including Alzheimer’s disease, chronic kidney disease, and cataract, which suggests that d-AAs may play a role in the process of aging (Fujii et al. 2011). In our previous study, we found strong correlations between d-AAs [d-asparagine (d-Asn), d-Ser, d-alanine (d-Ala) and d-proline (d-Pro)] in plasma and chronological age, clinical markers of organ dysfunction as well as markers of immune activation in both people living with HIV (PLWH), a disease known to be associated with accelerated/accentuated aging, as well as individuals without HIV between 25 and 84 years of age (Gabuzda et al. 2020; Yap et al. 2022). However, the pathophysiological role of plasma d-AAs in the context of human aging and inflammation is poorly understood.

d-Amino acid oxidase (DAO) and d-aspartate oxidase (DDO) are stereospecific degrative enzymes which metabolize d-AAs. DDO acts on acidic d-AAs, particularly d-aspartate, d-glutamate, and NMDA (Katane and Homma 2010) while non-polar d-AAs such as d-Ser and d-Ala, and hydrophobic and bulky d-AAs such as d-tyrosine, d-phenylalanine, and d-tryptophan are metabolized by DAO (Sacchi et al. 2018). Both DDO and DAO catalyze d-AAs to produce imino acid, coupled with the reduction of flavin adenine dinucleotide (FAD). Subsequently, FAD re-oxidizes spontaneously in the presence of oxygen to produce hydrogen peroxide (H_2_O_2_), while the imino acid hydrolyses to α-keto acid and ammonia (Sacchi et al. 2018).

H_2_O_2_ is a secondary messenger which regulates several signaling processes, such as cell proliferation, differentiation, and migration (Foreman et al. 2003; Li et al. 2006; Mailloux 2018; Ushio-Fukai 2006). H_2_O_2_ can have both positive and negative effects depending on the level of H_2_O_2_ and the cell type under investigation (Veal et al. 2007). H_2_O_2_ is also a reactive oxygen species (ROS) and at high levels causes oxidative stress, which initiates a number of downstream cascading events including mitochondria dysfunction, activation of pro-inflammatory cytokines, apoptotic and autophagic cell death; all pathways which have been shown to be involved in age-related organ decline (Finkel and Holbrook 2000; Gibson 2013; Pole et al. 2016; Redza-Dutordoir and Averill-Bates 2016).

Given our prior observation of age-related accumulation of plasma d-AAs and its association with increased immune activation (Yap et al. 2022), we posit that cellular exposure to d-AAs induces inflammation, mediated by the production of H_2_O_2_ and nuclear factor-κB (NF-κB) activation, leading to cell death. In this study, we investigated the in vitro effects of two abundantly found d-AAs in human plasma, d-Ser and d-Ala on human liver cancer cells (HepG2) and the downstream molecular mechanisms, contributing to d-Ser and d-Ala-induced inflammation.

## Methods

### Cell lines and cell culture

The human liver cancer cell line, HepG2 cell line, was purchased from ATCC (Virginia, USA) and was cultured in Eagle’s Minimum Essential Medium (EMEM; ATCC, Virginia, USA) supplemented with 10% fetal bovine serum (FBS), 100 U/mL penicillin and 100 µg/mL streptomycin (Gibco; Thermo Fisher Scientific, Massachusetts, USA) in a humidified incubator of 5% CO_2_ at 37 °C. Cells were subcultured by trypsinization at subconfluence and culture medium was changed every 2–3 days.

### Treatment of d-Ser and d-Ala

The concentrations of d-Ser and d-Ala used for all experimental assays were determined from the dose–response curves using the MTT (Sigma-Aldrich, St. Louis, MO, USA) assay. Briefly, cells were treated with increasing concentrations of d-Ala and d-Ser (Sigma-Aldrich, St. Louis, MO, USA) (12.5–600 mM) for 24 h and 48 h. 10 µL of MTT solution (5 mg/ml) was then added to each well containing 100 µL of culture medium, and the plates were incubated for 4 h at 37 °C. Formed formazan crystals were dissolved with 100 µL of isopropyl alcohol containing 0.05 N hydrochloric acid. Absorbance was measured at 570 nm using a microplate reader (BioTek, USA). The IC_50_ values, which represented the concentrations that inhibit 50% of cell growth, were obtained by plotting graphs of cell viability (%) against drug concentration (See supplementary Fig. 1 for dose–response curves). The IC_50_ values at 48 h, 30 mM, and 60 mM for d-Ser and d-Ala, respectively, were chosen as they were similar to concentrations used in previous studies (Brandish et al. 2006; Okada et al. 2017). Concentrations at half IC_50_, IC_50_, and two times of IC_50_ at 48 h were used for downstream experiments.

### RNA preparation and real-time reverse transcription-polymerase chain reaction (RT-qPCR)

HepG2 cells were seeded at approximately ~ 8 × 10^4^ cells/well in a 6-well tissue culture plate and grown until 80% confluence. Cells were treated with d-Ala and d-Ser for 48 h, trypsinized, and total cellular RNA isolated using Monarch^®^ Total RNA Miniprep Kit according to the manufacturer’s protocol (NEB, USA). 1 µg RNA was then reverse transcribed using LunaScript^®^ RT SuperMix Kit as per the manufacturer’s protocol. The *DAO* mRNA levels were determined by real-time PCR (ABI ViiA 7, Applied Biosystems, USA). The *DAO* primers used were: forward primer, 5′CGCAGACGTGATTGTCAACT′3; reverse primer, 5′GGATGATGTACGGGGAATTG′3. The reference gene beta-2-microglobulin (*B2M*) was used to normalize *DAO* mRNA levels and the sequences of the primers were: forward primer, 5′AGGACTGGTCTTTCTATCTCTTG′3; reverse primer, 5′CGGCATCTTCAAACCTCCAT′3. Thermal cycling was initiated with incubation at 50 °C for 2 min and 95 °C for 10 min followed by 40 cycles of 95 °C for 15 s, 60 °C for 1 min. Melt curve analysis was performed at the end of each PCR experiment. Normalized *DAO* mRNA levels were quantified by importing RT-qPCR quantification cycle (*C*_t_) values of the gene of interest and reference genes. Relative expression levels were calculated by ^ΔΔ^*C*_t_ methods (^Δ^*C*_t_ sample – ^Δ^*C*_t_ calibrator).

### Analysis of DAO and NF-κB proteins by Western blot

After 48 h of d-Ser and d-Ala treatment, cells were washed and lysed using cold RIPA buffer (Thermo Scientific, USA) containing 1 × protease and phosphatase inhibitor and 1 × EDTA for 15 min before scraping with a cell scraper. Lysates were centrifuged at 14,000×*g* for 15 min and supernatants were stored at − 80 °C until further analysis. The total protein concentration of the lysates was measured using Pierce™ BCA protein assay kit (Thermo Scientific, USA) and the amount of protein loaded in each well was standardized. The protein extracts were resolved in 10% SDS–polyacrylamide gel followed by western transfer to Immobilon polyvinylidene difluoride membrane (PVDF; EMD Millipore). Total protein on blot was stained using Pierce™ Reversible Protein Stain Kit (Thermo Scientific, USA). After blocking with 10% BSA in TBST for an hour, the membranes were incubated with the primary antibody at 4 °C overnight. The membranes were then washed and incubated with peroxidase-conjugated secondary antibodies for 1 h at room temperature. The antibodies used are as follows: rabbit anti-DAO (1:5000; Abcam), rabbit anti-pNF-kB (1:1000; Cell Signaling Technology), rabbit anti-NF-κB (1:1000; Cell Signaling Technology) and anti-rabbit IgG, horseradish peroxidase (HRP)-linked antibody (Cell Signaling Technology). Specific bands were visualized using the chemiluminescent Pierce™ ECL Western (Thermo Scientific, USA) and analyzed using GelAnalyzer 19.1. DAO protein levels were normalized to the total protein of the samples’ respective lanes. Relative protein expression was calculated and compared between control and treatment.

### Hydrogen peroxide measurement by Amplex Red assay

The production of H_2_O_2_ was measured using the Amplex Red kit (Molecular Probes/Invitrogen, USA). In brief, cells were seeded in 96-well microplates and left for 24 h for cell attachment and treated with d-Ala and d-Ser for 48 h. Amplex Red and HRP were added after 48 h and incubated at 37 °C for 60 min. The final concentrations of HRP and Amplex Red were 0.1 U/mL and 50 µM, respectively. The intensity was then measured in a multimode plate reader in fluorescent mode (SpectraMax M3, Molecular Devices, USA), with excitation at 540 nm and emission at 590 nm.

### Assessment of mitochondrial membrane potential

Changes in mitochondrial membrane potential (MMP) were detected with a lipophilic fluorochrome, JC-1 (BD™ MitoScreen, USA). Briefly, cells were seeded in 6-well plates and treated with d-Ala and d-Ser for 48 h after overnight serum starvation. The treated cells were detached using trypsin and pelleted. The cells were washed with PBS, incubated with JC-1 working solution for 15 min in an incubator with 5% CO_2_ at 37 °C and then washed twice with the assay buffer. The samples were analyzed by flow cytometry (BD FACSCanto II, USA), using the Green (FL-1) and Red (FL-2) channels, according to the manufacturer’s protocol.

### Measurement of TNF-α and IL-8 concentrations using Ella

Cell culture supernatants were collected after 48 h of d-Ser and d-Ala treatment. The levels of TNF-α and IL-8 were measured using an automated immunoassay system, Ella (Protein Simple, Bio-Techne, USA). Wash buffer was loaded into each designated well. The supernatants were diluted twofold with sample diluent and 50 µL of the diluted sample was added to each well of the 16 × 4 format cartridge. The cartridge was inserted into the Ella machine, and the analysis was performed using Simple Plex Runner 3.5.1.8 software (Bio-Techne, USA).

### Apoptosis assay and caspase activities

Apoptosis assay was done using FITC Annexin V Apoptosis Detection Kit (BD Pharmigen™, USA). Briefly, the HepG2 cell line was seeded at approximately ~ 250,000 cells per well in a 6-well plate and grown until 80% confluence. Cells were treated with d-Ala and d-Ser for 48 h after overnight serum starving. Cells were trypsinized, pelleted, and washed twice with cold PBS. The cells were then resuspended in 1 × Binding Buffer at a concentration of 1 × 10^6^ cells/mL. 100 µL of the suspension was then transferred to a 5 mL Falcon tube. FITC Annexin V and PI were added to the tube. The tube was mixed and incubated for 15 min at room temperature in the dark. The samples were analyzed by flow cytometry (BD FACSCanto II, USA) within an hour.

Caspases 8, 9, and 3/7 were measured using Caspase-Glo assay (Promega, USA) according to the manufacturer’s protocols. Briefly, the cells were seeded at a density of 5 × 10^3^ cells/well in 96-well white tissue culture plates. The cells were treated with d-Ala and d-Ser for 1, 3, 6, 9, 12, 18 and 24 h. The Caspase-Glo reagents were added to the wells and mixed well using a plate shaker at 400 rpm for 2 min. The plate was incubated at room temperature for 30 min. The luminescence of each sample was measured using a multimode plate reader in luminescence mode (SpectraMax M3, Molecular Devices, USA).

### Statistical analysis

Statistical analysis was performed using GraphPad Prism 8 (USA). All in vitro experiments were replicated at least 3 times. The data are presented as mean ± SEM and pairwise comparisons were performed using Student’s *t*-test. Statistical significance was defined as *p-*values less than 0.05.

## Result

### DAO mRNA and protein expression following d-Ser and d-Ala treatment

**Fig. 1:**
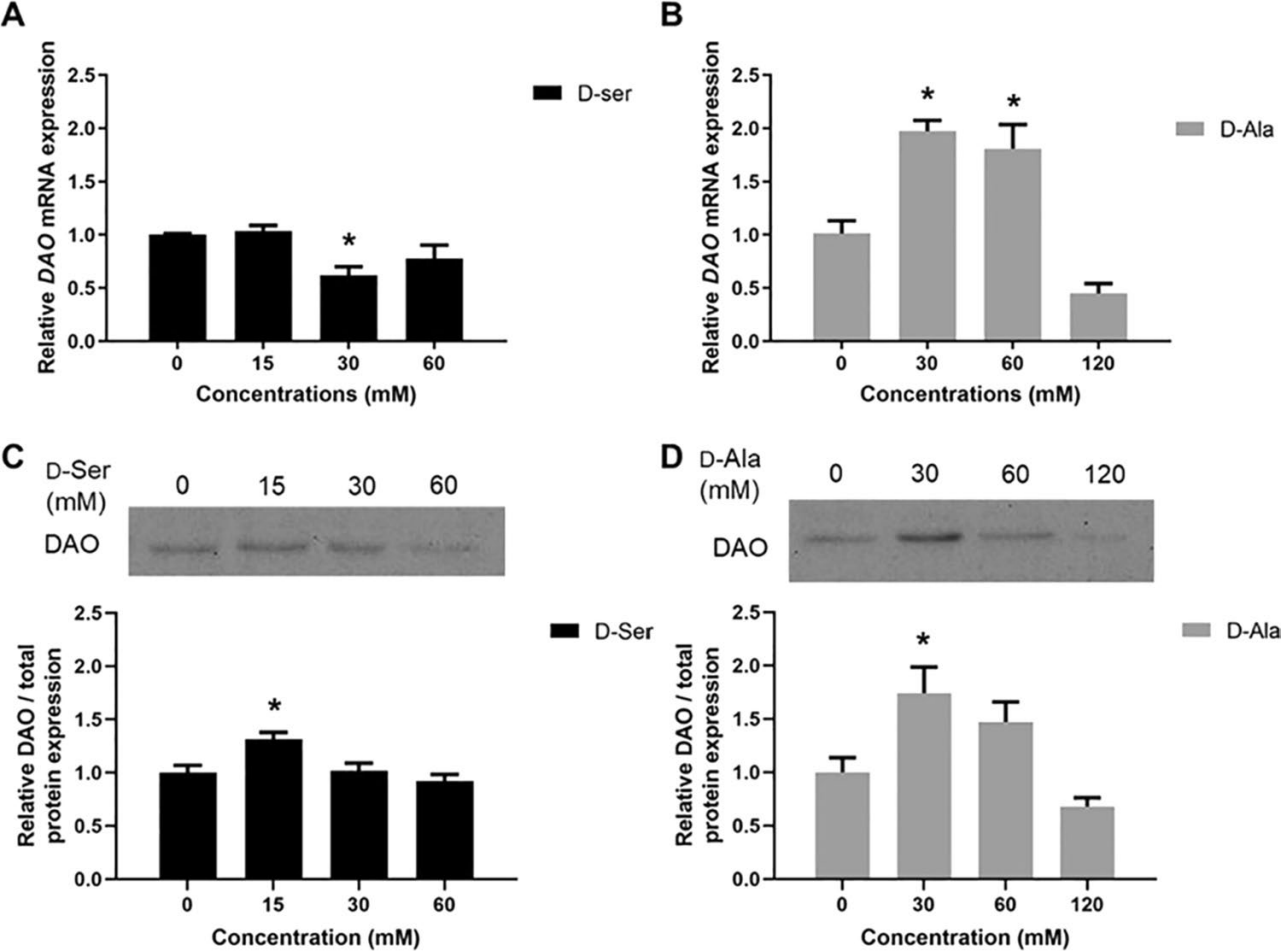
d-Ser treatment reduced *DAO* mRNA expression and induced DAO protein expression, whereas treatment of d-Ala increased *DAO* mRNA expression and DAO protein expression in HepG2 cells. Gene expression levels of *DAO* were measured using RT-qPCR following 48 h of d-Ser (**A**) and d-Ala (**B**) treatment. Representative western blots and relative quantitative analysis of DAO in d-Ser-treated (**C**) and d-Ala-treated (**D**) cells. The data are expressed as the mean ± SEM (*n* = 3). Data were compared by the Student’s *t*-test. *p < 0.05 as compared to untreated cells

DAO enzyme metabolizes neutral and non-polar d-AAs such as d-Ser and d-Ala. To examine the changes in *DAO* gene expression levels after d-Ser and d-Ala treatment on HepG2, RT-qPCR was performed to measure *DAO* mRNA expression. Figures 1A, B show the relative *DAO* mRNA expression after treatment with different concentrations of d-Ser and d-Ala. Contrary to expectation, the regulation of the *DAO* gene differed following exposure to d-Ser and d-Ala. The *DAO* mRNA expression was downregulated when cells were treated with higher concentrations of d-Ser. On the other hand, the gene expression of *DAO* in HepG2 cells increased by approximately twofold when treated with a low concentration of d-Ala and subsequently decreased with higher d-Ala concentrations.

We then investigated the level of DAO protein expression to confirm the changes in gene expression. A slight increase in DAO protein expression was observed in low concentrations of d-Ser treated cells. However, the protein expression remained unchanged when the cells were exposed to a higher concentration of d-Ser (Fig. 1C). For d-Ala, DAO protein expression concurred with the gene expression profile (Fig. 1D). These experiments provided the earliest indication that both entities likely had different molecular pathways, as we previously observed in our study on its effects in modulating immune activation (Yap et al. 2022).

### Hydrogen peroxide production following d-Ser and d-Ala treatment

**Fig. 2:**
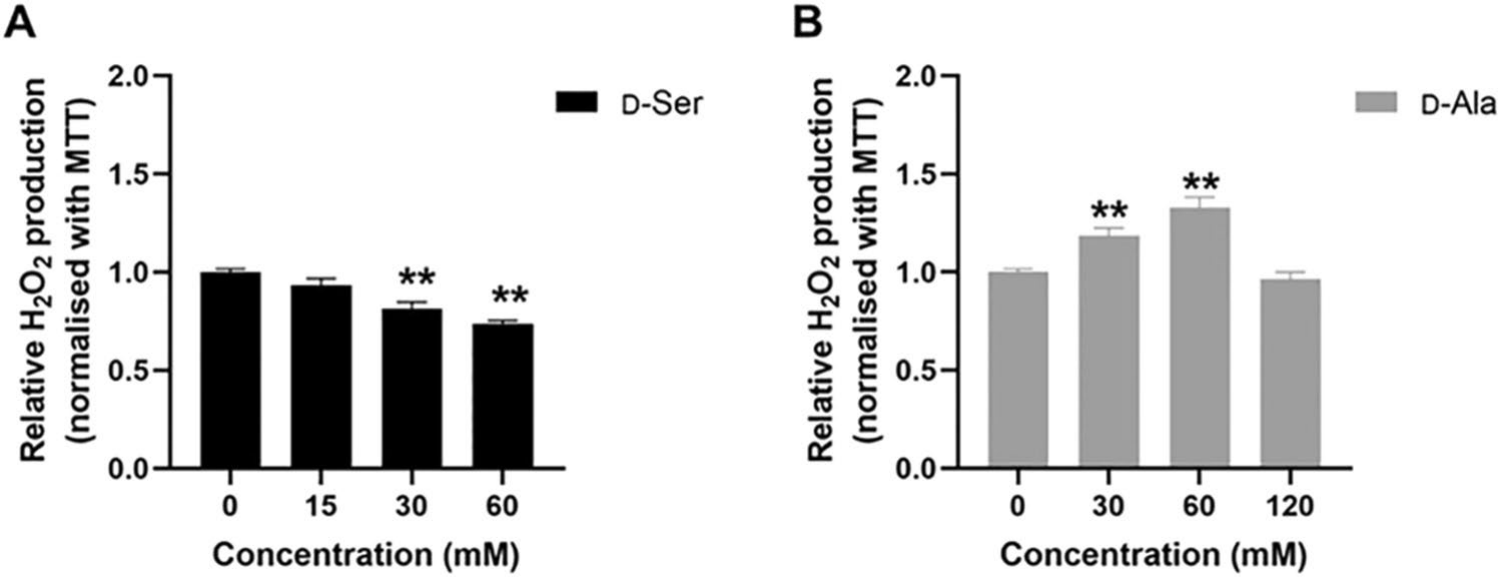
d-Ser treatment reduced hydrogen peroxide production but d-Ala treatment increased hydrogen peroxide production in HepG2 cells. The levels of hydrogen peroxide were measured by Amplex Red assay following 48 h of d-Ser (**A**) and d-Ala (**B**) treatment. The data are expressed as the mean ± SEM (*n* = 3). Data were compared by the Student’s *t*-test. **p < 0.001 as compared to untreated cells

DAO enzyme metabolizes d-AAs and produces H_2_O_2_ as a by-product. We next investigated the production of H_2_O_2_ following d-Ser and d-Ala treatment for 48 h using the Amplex Red assay. The assay employs an approach which converts Amplex Red to resofurin, a fluorescence compound, by HRP in the presence of H_2_O_2_. The concentration of H_2_O_2_ is reflected by the fluorescence intensity emitted by resofurin. Consistent with the profiles of *DAO* gene expression following exposure to different d-AA concentrations, we observed a reducing trend of H_2_O_2_ production in d-Ser-treated cells (Fig. 2A), whereas H_2_O_2_ levels were increased at lower concentrations of d-Ala but decreased at the highest concentration (Fig. 2B). This profile suggested that in cells treated with d-Ala, the increase in DAO enzyme led to the increased production of H_2_O_2_, whereas such metabolism did not occur in d-Ser-treated cells.

### Mitochondrial membrane potential (MMP) following d-Ser and d-Ala treatment

**Fig. 3:**
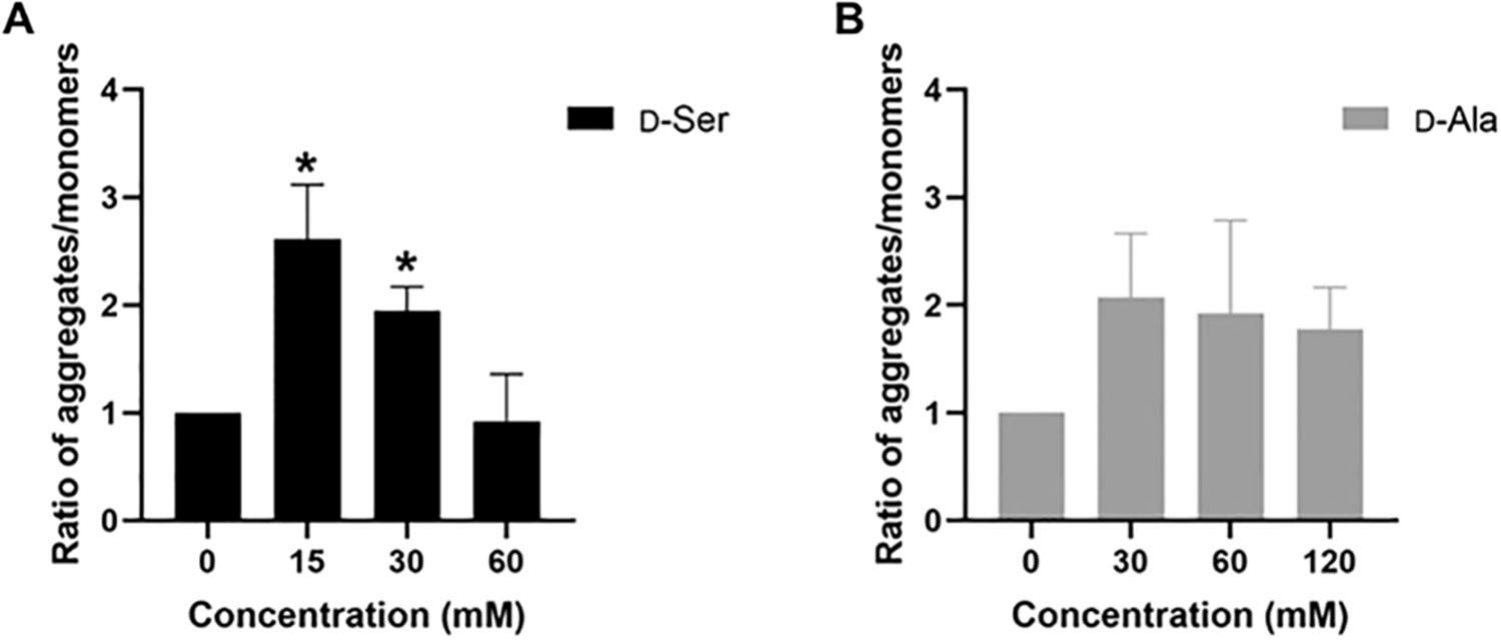
Mitochondrial membrane potential decreased after 48 h of d-Ser treatment but not in d-Ala-treated cells. The membrane potential was measured using the red/green fluorescence ratio of the JC-1 dye in the mitochondria following 48 h of d-Ser (**A**) and d-Ala (**B**) treatment. Red shift of the dye means more aggregates are formed (higher membrane potential; hyperpolarization), whereas a lower red to green ratio means few aggregates are formed (lower membrane potential; depolarization). The ratios of aggregates/monomers were calculated relative to untreated samples. The data are expressed as the mean ± SEM (*n* = 3). Data were compared by the Student’s *t* test. *p < 0.05 as compared to untreated cells

We then determined the effect of H_2_O_2_ production on MMP in HepG2 cells by employing JC-1 dye, as studies have shown that H_2_O_2_ decreases mitochondrial function, and subsequently initiates apoptosis (Li et al. 2003). JC-1 dye has been widely used to detect MMP in healthy and apoptotic cells across multiple cell types. JC-1 dye is a cationic dye exhibiting green fluorescence, which can permeate into the mitochondria where it accumulates, and forms reversible complexes called J aggregates in a concentration-dependent manner. Mitochondrial depolarization is indicated by a reduction in the aggregates to monomers ratio. d-Ser treatment led to hyperpolarization of mitochondria as reflected by a significant increase in the ratio of aggregates-to-monomers at low concentration but depolarized in a dose-dependent manner (Fig. 3A). A similar trend can be observed in d-Ala-treated cells, where hyperpolarization of MMP occurred at low concentration but depolarized when concentrations increased, but this did not reach statistical significance (Fig. 3B).

### Activation of NF-kB protein level and TNF-α and IL-8 concentrations in the supernatant following d-Ser and D-Ala treatment

**Fig. 4:**
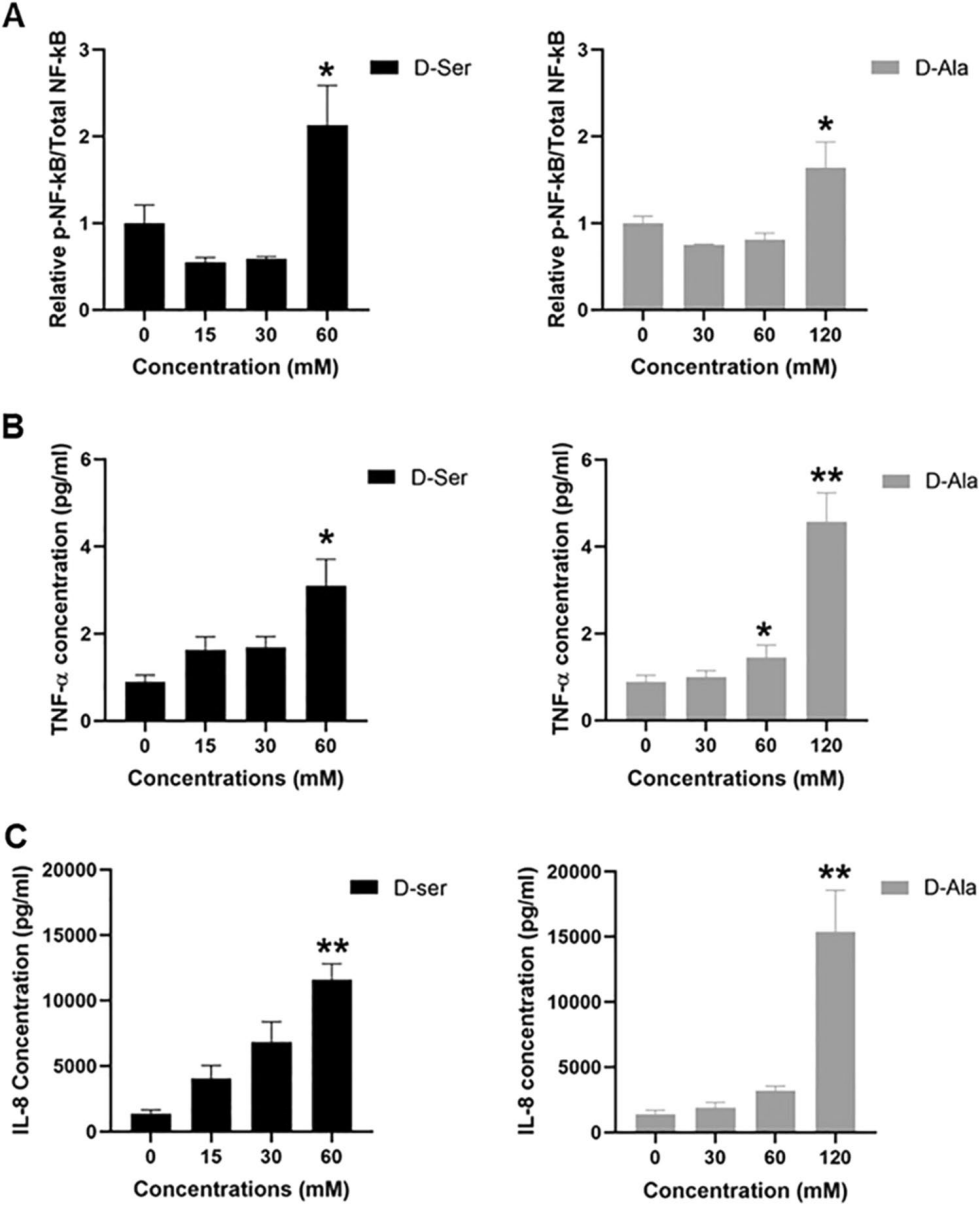
Phosphorylated NF-κB protein expression, TNF-α, and IL-8 concentrations in cell supernatant were increased at the highest concentrations of d-Ser and d-Ala treatment. The levels of phosphorylated NF-κB protein (**A**) were determined by Western blotting after 48 h of d-Ser and d-Ala treatment. The levels of TNF-α (**B**) and IL-8 (**C**) were determined by Ella after 48 h of d-Ser and d-Ala treatment. The data are expressed as the mean ± SEM (*n* = 3). Data were compared by the Student’s *t* test. *p < 0.05, **p < 0.001 as compared to untreated cells

H_2_O_2_ is involved in intracellular signal transduction pathways, including the activation of NF-κB (Chandel et al. 2000; Gunawardena et al. 2019). To investigate the role of NF-κB in d-AA metabolism, we measured the phosphorylated and total NF-κB in HepG2 cells following d-Ser and d-Ala treatment. We found that the level of phosphorylated NF-κB decreased at a lower concentration of d-Ser but increased by twofold at the highest concentration, a trend similarly seen in d-Ala-treated cells (Fig. 4A). Although we initially postulated that H_2_O_2_ was the likely stimuli of NF-κB activation, it appears that other pathways may also be involved particularly in the context of d-Ser.

NF-κB proteins play a critical role in regulating both innate and adaptive immune responses, and NF-κB activation controls gene expression of multiple inflammatory cytokines (Kimura et al. 2020). To confirm our prior in vivo findings (Yap et al. 2022), we measured the concentrations of pro-inflammatory cytokine, TNF-α and chemokine IL-8, in the cell supernatant after 48 h of d-Ser and d-Ala treatment using an automated ELISA platform. The result showed that higher concentrations of d-Ser increased the concentration of TNF-α, from 0.9 pg/ml in untreated to 3.1 pg/ml in 60 mM of d-Ser (Fig. 4B), which is similar to d-Ala treatment, from 0.9 pg/ml in untreated to 4.6 pg/ml in 120 mM of d-Ala (Fig. 4C). Furthermore, d-Ser treatment increased IL-8 concentration, from 1373.3 pg/ml in untreated to 11,597.7 pg/ml in 60 mM of d-Ser, whereas for d-Ala, from 1385.0 pg/ml in untreated to 15,388.3 pg/ml in 120 mM of d-Ala.

### Apoptosis assay and caspase assay

To examine if the production of H_2_O_2_ and TNF-α leads to apoptosis following d-Ser and d-Ala treatment, apoptosis was measured using annexin V following d-AA exposure. Annexin V binds strongly and specifically with phosphatidylserine, a phospholipid which exists in the inner leaflet of the plasma membrane during normal conditions. Cells that undergo apoptosis will transport phosphatidylserine from the inner to the outer leaflet of the plasma membrane, which signals the early stage of apoptosis. In conjunction with propidium iodide, annexin V binding assay can rapidly distinguish between apoptotic and necrotic cells.

**Fig. 5:**
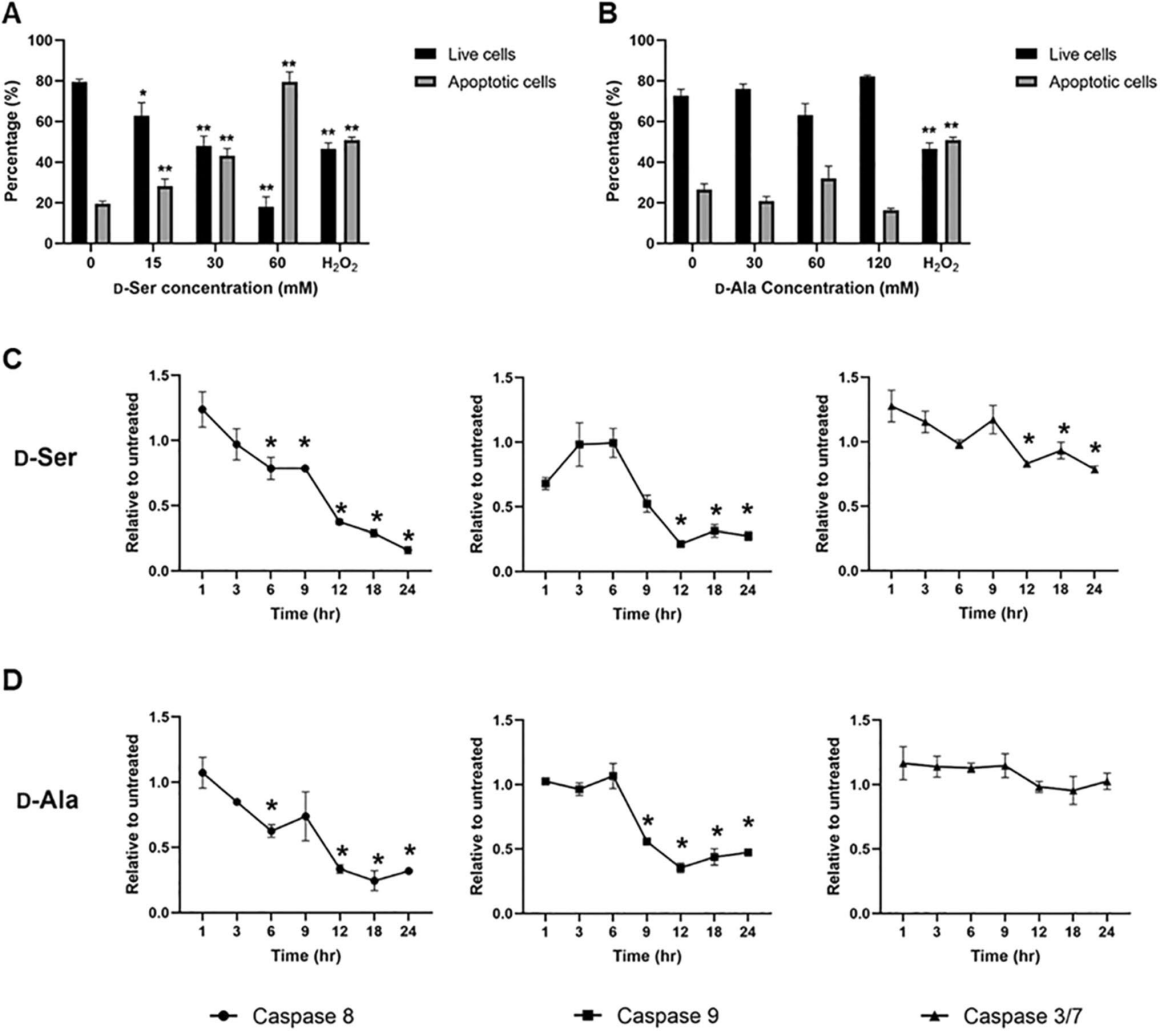
d-Ser induced a dose-dependent apoptosis in HepG2 cells but not d-Ala. Levels of caspase-8, 9, and 3/7 decreased in d-Ser treatment but only caspase-8 and 9 levels decreased in d-Ala treatment. Apoptosis was assessed using Annexin-V and propidium iodide (PI) kit after 48 h of d-Ser (**A**) and d-Ala (**B**) treatment using flow cytometry (see supplementary Fig. 3 for flow cytometry plots). Apoptotic cells were referred to as Annexin-V positive cells. Caspase-8, 9, and 3/7 activities were analyzed in HepG2 using Caspase-Glo kit over 24 h of d-Ser (**C**) and d-Ala (**D**) treatment. The data are expressed as the mean ± SEM (*n* = 3). Data were compared by the Student’s *t*-test. *p < 0.05, **p < 0.001 as compared to untreated cells

As shown in Fig. 5A, the percentage of apoptotic cells significantly increased following increasing treatment concentrations with d-Ser, where 50% of apoptotic cells were detected when cells were treated at IC_50_ of d-Ser and H_2_O_2_ (See Supplementary Fig. 2 for MTT assay for H_2_O_2_). In addition, the percentage of early apoptotic cells for all the concentrations was significantly increased compared to untreated cells, an observation also seen following H_2_O_2_ treatment, demonstrating that d-Ser induces apoptosis in a dose-dependent manner. Conversely, d-Ala treatment did not induce a dose-dependent apoptosis in HepG2, unlike d-Ser and H_2_O_2_ (Fig. 5B). These findings suggest that d-Ser may induce apoptosis by causing a collapse of MMP and high production of TNF-α and activated NF-κB, while d-Ala might trigger a cell survival signal with increased activation of NF-κB.

We subsequently measured caspase-8, 9, and 3/7 enzyme activity using a Caspase-Glo kit to identify whether apoptosis was caspase-dependent or caspase-independent. There was a significant reduction of caspase-8 after 6 h of incubation and caspase-9 after 9 h of incubation in both d-Ser- and d-Ala-treated cells (Fig. 5C, D). Caspase-3/7 was reduced after 12 h of d-Ser treatment over a 24-h time-period, whereas it remained unchanged in d-Ala-treated cells. We opted to measure the levels of caspases over the initial 24-h period before apoptosis occurred at 48 h as we reasoned that caspase activation precedes the process of apoptosis. These results demonstrated that treatment with d-Ser induced apoptosis in a caspase-independent manner, whereas there was no caspase activation in d-Ala-treated cells as it did not induce significant apoptosis.

## Discussion

**Fig. 6:**
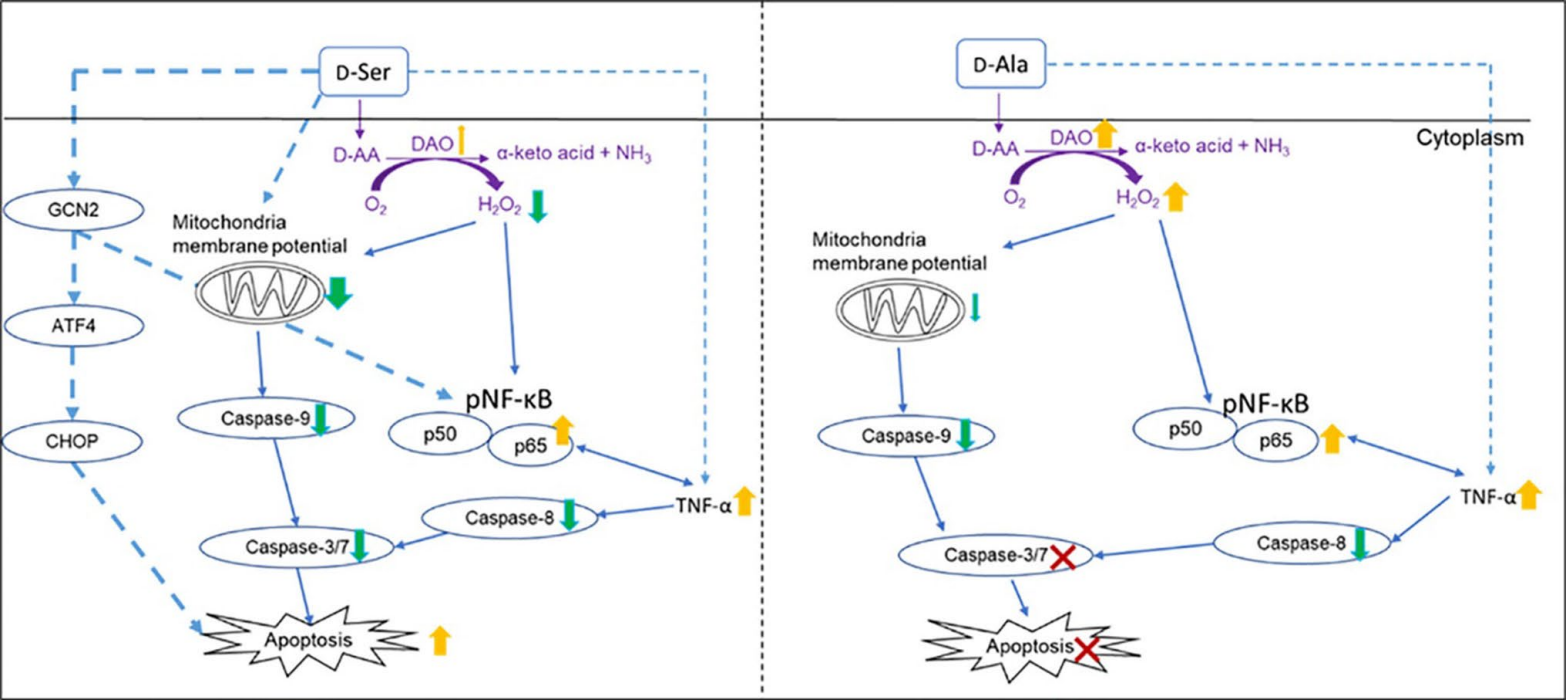
Schematic pathways for d-serine (d-Ser) and d-alanine (d-Ala) in HepG2 cells. In HepG2 cells, high concentrations of d-Ser activate general control nondepressible 2 (GCN2) instead of the DAO pathway, which induces the activation of NF-кB and the secretion of TNF-α, subsequently contributing to apoptosis. Although d-Ala did not cause apoptosis, the level of pro-inflammatory cytokine TNF-α increases through activation of NF-кB when HepG2 cells were treated with high concentrations of d-Ala

We previously reported that increased immune activation was correlated with elevated levels of plasma d-AAs (Yap et al. 2022). However, the cellular pathways following the breakdown of d-AAs by DAO in inducing inflammation have not been well studied. We hypothesized that the oxidation of d-AAs by the DAO enzyme produces high levels of H_2_O_2_ in cells, leading to oxidative stress and inflammation. Here, we explored the possible cellular mechanisms following exposure to increasing levels of d-Ser and d-Ala on HepG2 cells which express the *DAO* gene. We demonstrated that d-Ser and d-Ala affected cellular responses differently in HepG2 cells, albeit both leading to the production of inflammatory cytokines. When HepG2 cells were treated with d-Ser, *DAO* gene expression was downregulated, and protein expression was unchanged, with a decrease in H_2_O_2_ production and depolarization of MMP in a dose-dependent manner. Activated NF-κB levels increased at the highest concentrations of d-Ser, corresponding with high levels of TNF-α and IL-8 in cell culture supernatant. This led to a caspase-independent, dose-dependent apoptosis of cells. Contrary to the observations with d-Ser, the *DAO* gene and protein expressions were upregulated when HepG2 cells were treated with d-Ala, with increased production of H_2_O_2_ and depolarization of MMP at higher concentrations. This was accompanied by high levels of activated NF-κB, TNF-α and IL-8. No significant apoptosis was observed in d-Ala-treated cells. These findings suggest that treatment of d-Ser and d-Ala in HepG2 cells induces inflammation, possibly via distinct mechanisms (Fig. 6).

We mainly focused on d-Ser and d-Ala in this study as both are well-studied substrates of the DAO enzyme and are found in abundance as free forms in human plasma (Ishii et al. 2018; Miyoshi et al. 2011; Yap et al. 2022). d-Ser and d-Ala are also produced by intestinal microbiota and are suggested to have a potential role in the host-microbiome crosstalk in the context of aging (Matsumoto et al. 2018). HepG2 was selected as the representative model cell line for liver tissue, an organ which we found to demonstrate increased markers of liver fibrosis with the accumulation of plasma d-AAs in our prior study (Yap et al. 2022). Furthermore, HepG2 has been documented to have high *DAO* gene expression (Thul et al. 2017) and this facilitates our study of the effect(s)of d-AA naturally without having to depend on genetically modified models.

The concentrations of d-AAs in body fluid are maintained physiologically at low levels as detected in previous studies and the DAO enzyme is responsible for regulating neutral and non-polar d-AAs (Ishii et al. 2018; Kimura et al. 2020; Yap et al. 2022). The DAO enzyme is primarily located in the mammalian kidney, liver, and brain (Murtas et al. 2017). Although the exact mechanisms of DAO induction are not fully understood, it is likely that the substrates for DAO enzyme, including d-Ser and d-Ala, are one of the DAO inducers (Gabler and Fischer 1999). In this study, we observed that d-Ala upregulated *DAO* gene and protein expressions. On the other hand, treatment of d-Ser downregulated *DAO* gene expression, although there was a slight increase in DAO protein expression at low concentrations. This difference may potentially be attributed to differences in post-transcriptional regulatory mechanisms, including differences in the stability of mRNA or RNA processing or protein expression without a concurrent increase in gene expression (Buccitelli and Selbach 2020). Moreover, the DAO enzyme is known to metabolize both d-Ser and d-Ala but with a higher affinity for d-Ala (Murtas et al. 2017; Pollegioni et al. 2018). These results may indicate that the ability to induce DAO expression may differ between d-Ser and d-Ala, with d-Ala potentially being a stronger inducer compared to d-Ser in HepG2 cells. The levels of d-Ser are known to be tightly regulated by DAO enzyme in neurons and glial cells as d-Ser is a co-agonist for the NMDA receptor. Dysregulation of d-Ser levels affects the function of NMDA, which subsequently leads to Parkinson's disease and schizophrenia (Lu et al. 2011; Verrall et al. 2007). However, it is not well studied in hepatocytes.

The oxidation of d-AAs by the DAO enzyme produces H_2_O_2_, which acts as a secondary messenger in modulating normal cellular functions (Valko et al. 2007). Subsequently, we measured the levels of H_2_O_2_ and noted it decreased in d-Ser treated cells, while the production increased in d-Ala treated cells. These results are consistent with the results we obtained for *DAO* gene and protein expression, whereby H_2_O_2_ production correlated with increased DAO expression and vice versa. However, this finding was contrary to a previous study which showed that increasing concentrations of d-Ser produced an increasing amount of H_2_O_2_ in Chinese hamster ovary cells transfected with *DAO* gene (Brandish et al. 2006). This difference may be attributed to the elevated levels of DAO expression from the genetic modification of cells leading to higher amounts of H_2_O_2_ produced following d-Ser metabolism. Conversely, another study demonstrated that d-Ala increased free radical production in isolated liver mitochondria whereas d-Ser caused a much-muted response (Cortés-Rojo et al. 2007), which is consistent with our results. The difference in responses may also be attributed to the type of cells used where hepatocytes contain a variety of antioxidant and detoxifying enzymes which protect against oxidative stress and prevent damage to liver cells (Bardallo et al. 2022; Grant 1991), and thus may account for an attenuated H_2_O_2_ production.

H_2_O_2_ signaling is tightly regulated to maintain the intracellular redox homeostasis and the mitochondrial ROS scavenging system is responsible to remove excess H_2_O_2_ and superoxide (Kusama et al. 1989; Zhou and O’Rourke 2012). However, unregulated H_2_O_2_ production which overwhelms the scavenging system will cause MMP depolarization and eventually lead to mitochondrial failure and cell death (Richardson and Schadt 2014). Treatment with H_2_O_2_ was found to decrease MMP in a dose-dependent manner, causing necrosis, as well as caspase-dependent apoptosis in lung cancer cells (Park 2018). Studies have also found that an increase of MMP triggered by low concentrations of etoposide in HL60 cells to correlate with the cell cycle arrest, whereas increasing concentrations of etoposide induces massive apoptosis and a collapse of MMP (Facompré et al. 2000). In our study, we observed that d-Ser decreased MMP in a dose-dependent manner, despite the reduction of H_2_O_2_ production. Similar to d-Ser, there was a slight depolarization of MMP in d-Ala-treated cells with increased concentrations, although not statistically significant. These results suggest d-Ser may impact MMP by means other than H_2_O_2_, which could include elevated ATP production or disruption of various macromolecules within the mitochondria, resulting in altered mitochondrial function (Logan et al. 2016; Sakamuru et al. 2022). Conversely, d-Ala had a lower effect on MMP despite increased levels of H_2_O_2_, possibly due to the detoxifying capacity of hepatocytes, a natural function of these cells (Bardallo et al. 2022). These findings collectively suggest that d-AAs exposure triggers varying degrees of cellular signaling, and this is dependent on the specific type of d-AA and its concentration.

H_2_O_2_ can trigger the NF-κB transcription factor, by phosphorylating IκBα at Tyr42 which leads to IκBα dissociation from NF-κB and its eventual degradation (Perkins 2007). The activation of NF-κB protein promotes the expression of pro-inflammatory cytokines, TNF-α, IL-6, IL-1β, and chemokine, IL-8 (Liu et al. 2017; Yamamoto and Gaynor 2004). This physiological signaling process is a key stress response signaling pathway, which is also a significant pathophysiological feature of aging (Papaconstantinou 2019). The data from this present study suggest that both d-Ser and d-Ala may have a pro-inflammatory role through activation of NF-κB when exposed to high concentrations. This finding is consistent with that of Okada et al. (2017) who discovered that in the human tubular cells, d-Ser induced the secretion of pro-inflammatory cytokine, IL-6, and chemokine, IL-8 (Okada et al. 2017). Furthermore, d-Ala was suggested to be recognized by mammalian hosts as a bacterial signature, like a pathogen- or microbe-associated pattern, to stimulate immune responses (Suzuki et al. 2021). The activation of NF-κB by d-Ala is likely through DAO-induced H_2_O_2_ as observed in our study, whereas d-Ser through alternative pathways. However, we note that the increase of NF-кB activation occurred only at high d-Ala concentrations and not at lower concentrations consistent with H_2_O_2_ production following d-Ala exposure. We speculate that this may be due to physiological or other compensatory mechanisms at play which initially attenuate the activation of NF-кB but eventually become overwhelmed beyond a certain threshold, a concept previously described with the interaction of H_2_O_2_ and insulin signaling (Iwakami et al. 2011). Further studies assessing oxidative and inflammation gene/protein arrays may be useful to discern this. It is also noteworthy that NF-кB activation was observed at high concentrations of d-AAs in this study. We are currently uncertain about the highest physiological concentrations achieved by d-AAs as this has not been reported. d-AAs were found to accumulate in aged tissues and age-related diseases (Chervyakov et al. 2011; Fujii et al. 2011). It is possible that the enzymatic function of DAO to become saturated and for d-AAs to accumulate triggering various biological pathways like NF-кB. Though this accumulation may not be as high as twice IC_50_ as used in this study, it does demonstrate that high levels of d-AAs are immunogenic.

The overproduction of H_2_O_2_, loss of MMP, and high levels of TNF-α may ultimately lead to apoptosis, a programmed cell death (Li et al. 2003; Xiang et al. 2016; Micheau and Tschopp 2003). Apoptosis can be initiated by two signaling pathways, caspase-independent and caspase-dependent. Caspase-dependent apoptosis is triggered through both extrinsic and intrinsic pathways. The extrinsic pathway involves transmembrane receptor-mediated interactions, such as death receptors which are members of the TNF-receptor gene superfamily, and result in the activation of the caspase-8 enzyme (Redza-Dutordoir and Averill-Bates 2016; Tummers and Green 2017). The intrinsic pathway involves a diverse array of non-receptor-mediated stimuli that produce intracellular signals that act directly on targets within the cell and are mitochondrial-initiated events, and this activates the caspase-9 enzyme (Redza-Dutordoir and Averill-Bates 2016). Both extrinsic and intrinsic pathways eventually activate the major effector caspases, caspase-3, and caspase-7, leading to apoptosis. We observed a dose-dependent, caspase-independent apoptosis in d-Ser-treated cells, while no caspase activation or significant apoptosis in d-Ala-treated cells. A previous in vitro study investigating the underlying mechanisms of d-Ser in kidney cell lines, HK-2 and NHREC, which do not express the *DAO* gene, demonstrated that d-Ser induced apoptosis and cellular senescence through the DAO-independent general control nondepressible 2 (GCN2) pathway (Okada et al. 2017). This pathway is activated when amino acid starvation occurs and it induces apoptosis and activation of NF-кB (Jiang et al. 2003; Kilberg et al. 2012). The study also demonstrated an increase in caspase-3/7 activity after exposure to d-Ser, which is contrary to our finding. A possible explanation for this discrepancy could be the differential response mechanisms of kidney cells and liver cells to d-Ser. It is conceivable that in liver cells, d-Ser induces a caspase-inhibitory condition, resulting in the caspase being in the inactivated state and unable to initiate apoptosis and potentially leading to a shift towards necrosis (Lou et al. 2021). It was demonstrated that the presence of positive annexin V and negative propidium iodide signal might also indicate the presence of necrotic cells (Sawai and Domae 2011). However, further investigation is warranted to delve into the intricate mechanisms underlying the differential response observed in specific cell lines following d-Ser treatment. A previous study found that d-Ala did not exert any toxicity towards tubular cells, which is consistent with our result, even though the concentrations we used in our study were higher and we employed a different cell line (Okada et al. 2017). A possible explanation is that a high level of H_2_O_2_ triggers the activation of NF-кB, which may act as a pro-survival signal instead of apoptosis. The NF-кB activation subsequently induces the transcription of pro-inflammatory cytokines including TNF-α, which can also act as a molecular switch that induces inflammation and cell survival (Kaminskyy and Zhivotovsky 2014; Sauer et al. 2000). These results may imply that the cascading mechanism induced by d-Ala may act as a pro-survival signal instead of apoptosis, unlike d-Ser. Nevertheless, our understanding of the biological consequence of d-Ala on HepG2 is currently limited.

Taken together, we demonstrated that both d-Ser and d-Ala induced inflammation in HepG2 cells. This inflammation, in conjunction with mitochondria dysfunction, intensifies the apoptosis signal in d-Ser-treated cells, through a DAO-independent pathway. Despite both entities inducing elevated NF-κB levels, d-Ala-treated cells may receive that signal as a pro-survival signal rather than promoting cell death. These results provide additional insights into the distinct effects of d-Ser and d-Ala in HepG2 cells, highlighting their involvement in separate pathways and the magnitude of the signal that is being triggered. Further molecular investigations are necessary to gain a more comprehensive understanding of the underlying mechanisms responsible for d-Ser- and d-Ala-induced inflammation and their subsequent cellular consequences.

## Supplementary Information

Below is the link to the electronic supplementary material.

Supplementary file1 (DOCX 465 KB)

## Data Availability

The data that support the findings of this study are available from the corresponding author upon reasonable request.
