# Supplementary material for: d-Amino acids differentially trigger an inflammatory environment in vitro
Source: Amino Acids. 2024 Feb 3;56(1):6. doi: 10.1007/s00726-023-03360-8 (PMC10838247; doi:10.1007/s00726-023-03360-8)
Supplement: Supplementary file 1 — Supplementary file1 (DOCX 465 KB) [file 726_2023_3360_MOESM1_ESM.docx]

**Supplementary information**


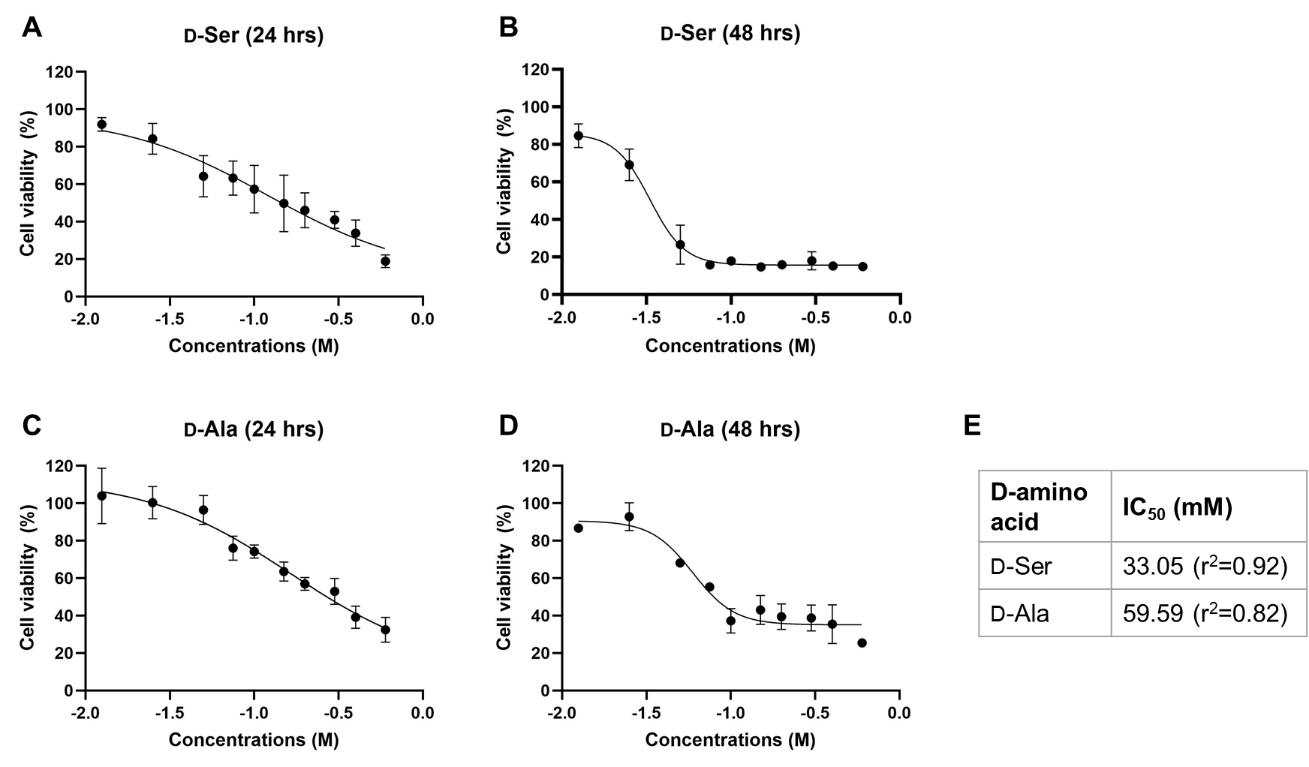


Supplementary Figure 1: Dose-response curve of D-Ser (A, B) and D-Ala (C, D) on HepG2 cells over a 24- and 48-hrs period using the MTT assay. HepG2 were treated with various concentrations (ranging from 12.5 mM to 600 mM) for 24 and 48 hrs. Increasing concentrations of D-Ser and D-Ala reduced the cell viability of HepG2. The data are expressed as the mean ± SEM (n = 3).


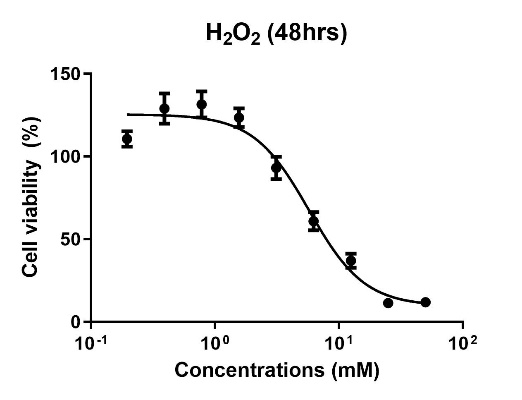


Supplementary Figure 2: Dose-response curve of H_2_O_2_ on HepG2 cells over a 48-h period using the MTT assay. Increasing concentrations of H_2_O_2_ reduced the cell viability of HepG2 and the calculated IC_50_ for H_2_O_2_ is approximately 5.8 mM. The data are expressed as the mean ± SEM (n = 3).


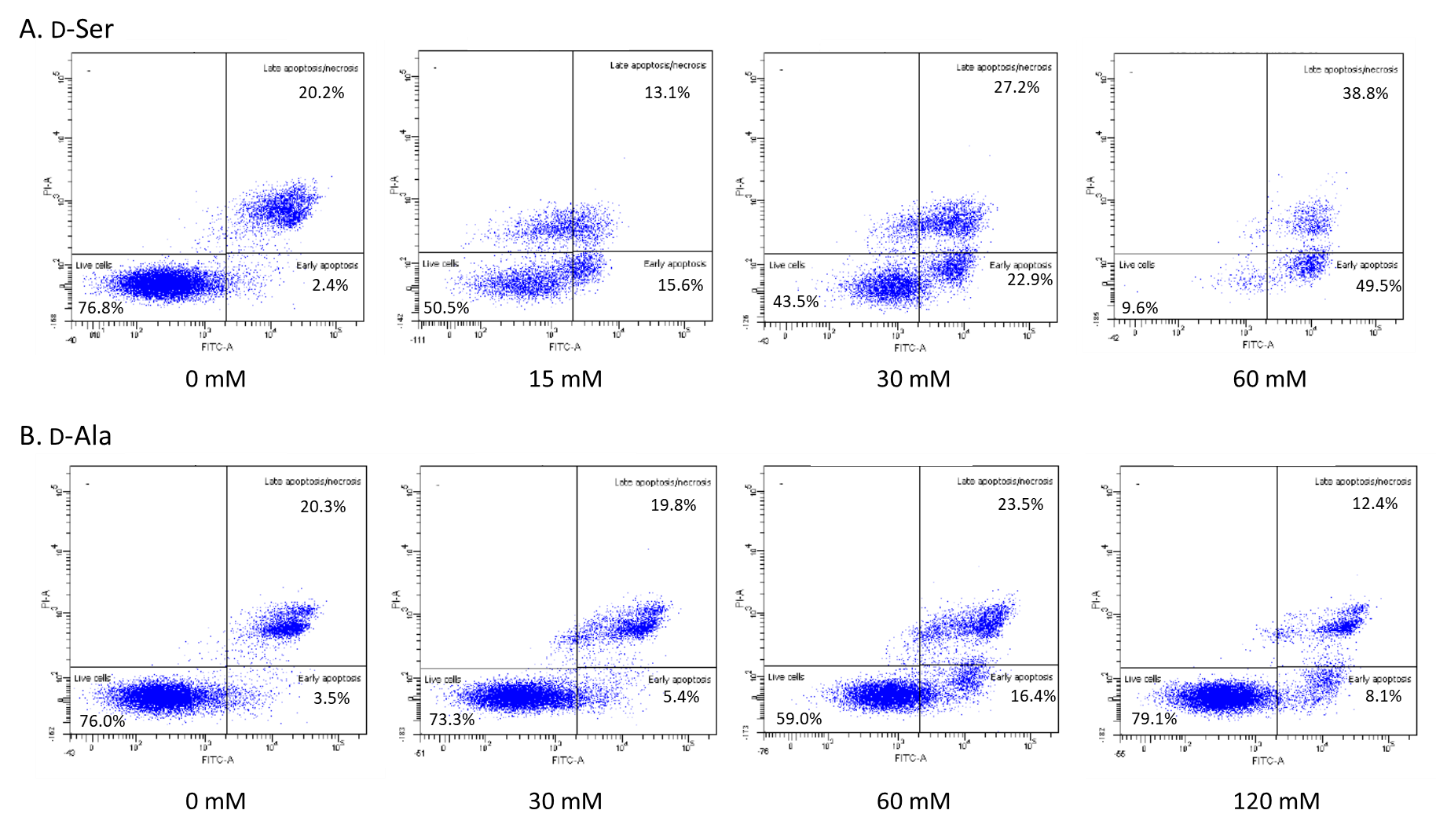


Supplementary Figure 3: Flow cytometry plots for apoptosis using Annexin-V (FITC) binding and propidium iodide (PI) staining in HepG2 cells. The plots shown represent one of the experiment replicates.
